# Current and Emerging Therapies for Targeting Protein Arginine Methyltransferases (PRMTs) in Cancer

**DOI:** 10.3390/ijms26167907

**Published:** 2025-08-16

**Authors:** Adriana Kaganovski, Bayle Smith-Salzberg, Hadar K. Shimshon, Andrew Draheim, Mark Spivak, Tzuriel Sapir, David Shifteh

**Affiliations:** 1 College of Medicine, SUNY Downstate Health Sciences University, Brooklyn, NY 11203, USA; adriana.kaganovski@downstate.edu (A.K.); bayle.smith-salzberg@downstate.edu (B.S.-S.); hadar.shimshon@downstate.edu (H.K.S.); andrew.draheim@downstate.edu (A.D.); mark.spivak@downstate.edu (M.S.); 2 Perelman School of Medicine, University of Pennsylvania, Philadelphia, PA 19104, USA

**Keywords:** PRMT, PRMT1, PRMT2, PRMT3, PRMT4, PRMT5, PRMT6, PRMT7, PRMT8, PRMT9

## Abstract

Protein arginine methyltransferases (PRMTs) are a class of enzymes that mediate critical post-translational modifications through arginine methylation as epigenetic regulators. PRMTs have been shown to have a vast array of regulatory effects including in gene expression, signal transduction, and cellular proliferation. Dysregulation of PRMT activity has been seen in the progression of various cancers, including breast, lung, and colorectal cancer. Moreover, PRMT overexpression has been shown to correlate with poor patient prognosis. This review aims to explore the roles of the individual PRMTs in cancer and aims to highlight the latest and newest developments of PRMT inhibitors as emerging therapeutic strategies. Numerous preclinical and clinical studies have identified several novel compounds that effectively target PRMT activity and have shown significant therapeutic results. As such, this review aims to not only highlight the current research findings, but to also emphasize the significant need for future research on PRMTs as novel therapeutic targets in cancer.

## 1. Introduction

In recent years there has been an increased understanding of cancer as being caused not only by genetic alterations, but also by epigenetic alterations and post-translational modifications. Over this time there has been a growing body of evidence indicating that post-translational modifications play an important role in cancer progression and maintenance [1].

Methylation of arginine residues is one such type of post-translational modification which has broad cellular effects including altering gene expression, protein modification, signal transduction, and cell cycle progression. Arginine methylation is catalyzed by a group of enzymes termed protein arginine methyltransferases (PRMTs). PRMTs use S-Adenosyl methionine (AdoMet/SAM) as a cosubstrate and methyl donor for the transfer of methyl groups to arginine residues of histone and non-histone proteins [2].

PRMTs are classified based on the type of methylation they perform. When two methyl groups are placed on one of the terminal nitrogen atoms of the guanidino group, it leads to the formation of the asymmetric dimethylarginine derivative (ADMA). When one methyl group is placed on each of the two terminal guanidino nitrogen atoms, it produces the symmetric dimethylarginine derivative (SDMA), and when a single methyl group is placed on one of the terminal nitrogen atoms, it is known as the monomethylated arginine derivative (MMA) [3]. Nine PRMTs have been classified so far. The type I PRMTs, which catalyze the formation of ADMA and MMA, consist of PRMT1, PRMT2, PRMT3, PRMT4, PRMT6, and PRMT8. The type II PRMTs, which form SDMA and MMA, consist of PRMT5 and PRMT9. PRMT7 is the sole type III PRMT and forms MMA [3]. An overview of the mechanism of action of the PRMT family members can be seen in Table 1 below.

PRMTs are overexpressed in a number of cancers including melanoma, multiple myeloma, glioblastoma, lung, bladder urothelial, gastric, cervical, ovarian, and colorectal cancers, and dysregulation of PRMTs is closely associated with poor patient prognosis [3,4,5,6,7,8,9,10,11,12,13]. As such, PRMT targeting has garnered significant interest as a novel approach for anticancer drug development [14,15]. In light of these findings, several PRMT inhibitors are currently being tested, both in labs and clinics, as a potential therapeutic option in several cancer types.

PRMTs sit alongside other epigenetic regulators, including DNA methyltransferases (DNMTs) that methylate DNA; histone lysine methyltransferases/demethylases (KMTs/KDMs) that write/erase lysine methyl marks; histone acetyltransferases/deacetylases (HATs/HDACs) that add/remove acetyl groups; ubiquitin/SUMO modifiers; and ATP-dependent chromatin remodelers. Like these enzymes, PRMTs act as “dimmer switches” for gene programs, sometimes cooperating or competing with other marks (e.g., arginine methylation can oppose or reinforce lysine methylation or acetylation at the same promoter). What differentiates PRMT inhibition is its strong reach beyond chromatin—into RNA processing and immune signaling—creating distinct vulnerabilities and biomarker strategies, while still overlapping with other epigenetic drugs on common cancer pathways. In the big picture, PRMTs are nodal integrators that connect chromatin state to RNA splicing and signaling, which explains both their unique therapeutic potential and their rationale for combination with DNMT, HDAC, BET, DNA-damage, or immunotherapy approaches [1,2,3,4,5,6,7,8,9,10,11,12,13,14,15].

In this review article, we examine the latest evidence for the role of each of the PRMTs in the development of cancer. We also explore the efficacy and clinical utility of PRMT inhibitors. Finally, we provide an overview of emerging cancer therapies which target each of the PRMTs in cancer. An overview of the cancer-relevant roles of the PRMT family members can be seen in Table 2 below.

## 2. Protein Arginine Methyltransferases (PRMTs)

### 2.1. PRMT1

Protein arginine methyltransferase 1 (PRMT1) has been documented as a regulator across multiple biological processes, encompassing RNA metabolism, maintenance of genome stability, and signal transduction [16]. Its dysregulation, typically upregulation, contributes to the pathogenesis of cancer [17]. Notably, in hematological and solid tumors such as lung cancer, PRMT1 fosters tumor growth [18]. Additionally, the concurrent upregulation of PRMT1 expression and methylation of the Arg342 residue of EZH2 correlates significantly with adverse clinical prognoses among breast cancer patients [16]. Due to the recognition of PRMT1’s significance as an oncogenic protein, innovative therapeutic interventions are currently in use in clinical trials. AMI-1, discovered in 2004, inhibits type 1 PRMTs, effectively reducing the proliferation of tumor cells and actively inducing apoptosis [16,19]. Allantodapsone, a pan-inhibitor of S. aureus, is specific for PRMT1, inhibiting activity at an identical efficiency to that of AMI-1 [16,20]. MS023, an additional PRMT1 inhibitor undergoing clinical trials, demonstrates efficacy in surpassing the requisite threshold to trigger apoptosis in cancer cells [16,21]. In solid tumor malignancies, Type 1 PRMT inhibitor GSK3368715 has been shown to regulate tumor activity via shifting arginine methylation states [22].

Recent developments targeting PRMT1 have also shown promise in advantageously regulating the type I protein arginine methyltransferase in specific cancer treatments. MS023 and GSK3368715 were found effective in blocking methylation of the cGAS/STING DNA complex, inhibiting tumor cell evasion in solid malignancies [23]. In breast cancer, GSK3368715 was effective in treatment when combined with chemotherapies such as cisplatin and camptothecin, and in conjunction with erlotinib [24]. In leukemias, Type I inhibitors can be used to block disease-associated splenomegaly [25]. Arginine methylation appears to be a powerful target when treating cancer, yet more research needs to be carried out on the specific regulatory mechanisms behind PRMT1 to develop a widely successful inhibitor.

### 2.2. PRMT2

Protein arginine methyltransferase 2 (PRMT2), like PRMT1, is a Type 1 PRMT. Therefore, PRMT2 is involved in the formation of asymmetric dimethylarginine [26]. PRMT2 is also largely involved in the processes of oncogenesis. It has been demonstrated that PRMT2 interacts with the retinoblastoma protein (Rb), modulating its activity via the E2F transcription factor [27]. Additionally, PRMT2 promotes apoptosis by inhibiting NF-kB-dependent transcription, facilitated by its SAM-binding domain interacting with IkB-a through its ankyrin domain [26]. However, because PRMT2’s methyl transferase activity is difficult to measure enzymatically, it is one of the least studied methyltransferases [26]. Nevertheless, recent studies show promise in mediating cancer progression through the inhibition of PRMT2. 

PRMT2 is a novel target for new therapeutic therapies in cancer treatment. In renal cell carcinoma (RCC), PRMT2 promotes cancer cell proliferation and migration, while also activating WNT transcription. These findings positively correlate with poor prognosis in cancer patients [28]. In hepatocellular cancer cells, inactivation of PRMT2 inhibited cell growth and induced apoptosis through the blockage of Bcl2 [29]. PRMT2 regulates breast cancer progression as well, the loss of the protein leading to increased cyclin D1 expression, resulting in cancer progression [30]. When PRMT2 is downregulated in breast cancer, tamoxifen, a commonly used selective-estrogen receptor modulator, is rendered ineffective as well [31]. Additionally, in glioblastomas, PRMT2 has been identified as a pro-tumorigenic factor by modifying both promoters and enhancers, upregulating oncogenic transcription [32]. While PRMT2 does regulate cell growth, silencing of the protein does not bring cancer cells to apoptosis [32]. Therefore, a greater understanding of the mechanisms behind PRMT2 is needed before an effective therapy is developed.

### 2.3. PRMT3

PRMT3’s physiological role in the body is to regulate ribosomal maturation and homeostasis via methylation of ribosomal protein S2 (rpS2), which is crucial to the maturation of the 80S ribosome, and via methylation of treacle ribosome biogenesis factor 1 (TCOF1) at R399, which regulates ribosomal homeostasis [33]. In addition, PRMT3 has been shown to be involved in osteogenic homeostasis by inducing the differentiation of mesenchymal stem cells into osteoblastic cells capable of bone development [34]. These diverse roles of PRMT3 in regulation of protein translation and osteogenic activity highlight the clinical importance of developing therapeutic agents for pathologies arising from mutations in these processes.

PRMT3 has been implicated in numerous malignancies. PRMT3 has been shown to have an increased expression in patients with glioblastoma, with in vitro studies showing that PRMT3 expression is necessary for glioblastoma cell proliferation and invasive metastasis [35]. PRMT3 has also been shown to be overexpressed in colon cancer tumors, possibly due to its role in regulating c-MYC polyubiquitination and stabilization. PRMT3 has also been correlated with a lower survival rate in patients with colon cancer compared to colon cancer patients with lower expression of PRMT3 [36]. Finally, PRMT3 has been shown to have a greater than two-fold increase in expression in pancreatic ductal cells from patients with pancreatic cancer [37]. These studies indicate that PMRT3 inhibitors are a possible treatment for these specific cancers.

The potent PRMT3 inhibitor SGC707 has been shown to have potential therapeutic value in multiple studies. In one study, it had been shown to inhibit the differentiation of mesenchymal stem cells into osteoblastic cells by allosterically inhibiting the protein’s methyltransferase activity [34]. Additionally, combined therapy by anti-PD-1 and SGC707 has been shown to be more effective in inducing ferroptosis in endometrial cancer cells than treatment with anti-PD-1 alone, possibly due to SGC707 suppressing resistance to ferroptosis in these cells [38]. Finally, one study showed that increasing concentration of SGC707 were effective at decreasing the asymmetric dimethylarginine modification of lactate dehydrogenase A by PRMT3 in hepatocellular carcinoma, dampened any increases in glycolysis in PRMT3 upregulating cells, and mediated and weakened the proliferation capacity of these cells [39]. Another study identified additional potent inhibitors of PRMT3 labeled compounds 29, 30, 36, and 37, which act similarly to SGC707 (referred to as compound 4 in this study) [40]. These lab-tested inhibitors of PRMT3 warrant further exploration clinically as they relate to the treatment of PRMT3 associated malignancies.

### 2.4. PRMT4

PRMT4, also known as CARM1, has diverse biological roles which include inducing embryologic blastomere cells to differentiate into cells of the inner cell mass via the activation of the transcription factors Nanog, Sox21, Sox2, and Oct4, the latter two via the direct result of methylation of histone H3 by PRMT4 [41]. Additionally, studies have shown that PRMT4 has a role in T-cell maturation via methylation of thymocyte cAMP-regulated phosphoprotein, is a coactivator of PPARγ, a transcription factor necessary for adipocyte development, methylates the transcription factor SOX9 to control cartilage development and subsequent endochondral ossification, plays a role in maintenance of normal lung epithelium, and plays a role in cardiac tissue maturation [42].

PRMT4 has been shown to play an active role in numerous cancers. In hepatocellular carcinoma, PRMT4 exerts its effects by activating the AKT/mTOR pathway. This method of action was proven via an experiment which resulted in a reduction in cells which overexpressed PRMT4 using MK2206, an AKT inhibitor, and rapamycin, an mTOR inhibitor [43]. These results show a promising therapeutic use of AKT/mTOR inhibitors in PRMT4 induced hepatocellular carcinoma. PRMT4 has also been shown to play a role in acute myelogenous leukemia by inhibiting differentiation of human stem/progenitor cells. It does so by methylating the transcription factor RUNX1 at R223. Likewise, in vitro studies which knocked out PRMT4 induced differentiation whereas in vivo studies also induced apoptosis of myeloid leukemic cells [44]. These results show a promising area of research regarding the clinical application of PRMT4 inhibition in the treatment of patients with acute myelogenous leukemia.

Numerous inhibitors of PRMT4 have been developed as promising cancer therapeutics. One of these inhibitors is TP-064, which when tested on various cancer cell lines, demonstrated anti-proliferative effects on multiple myeloma cells [45]. Given the substantial body of research underscoring the critical role of PRMT4 in oncogenesis, multiple teams have successfully engineered targeted inhibitors against PRMT4 [46]. These developments merit further investigation to evaluate their efficacy as inhibitors, as they have the potential to deliver much-needed advancements in cancer treatment for cancer patients. Additionally, a potent, selective inhibitor of PRMT4 called compound 49 was tested in multiple cancer cell lines and showed a notable antiproliferative effect on the MOLM13 acute myeloid leukemic cell line. Follow up experiments using compound 49 showed a direct inhibition of PRMT4’s asymmetric dimethylation function [47]. These studies highlight the urgent need for further research and development of PRMT4 inhibitors as they pertain to the treatment of cancer patients.

### 2.5. PRMT5

PRMT5 is a type II arginine methyltransferase that plays an important role in the G1-S cell cycle progression, cytokine signaling, migration, and maintenance of the pluripotent embryonic neural stem cells [14]. Additionally, PRMT5 maintains iNK, CD4^+^, and CD8^+^ populations, as well as their survival and proliferation [34]. PRMT5 protein exhibits a tertiary structure characterized by a triose-phosphate isomerase (TIM) barrel at the N-terminus. This structure ultimately interacts with the methylosome protein 50 (MEP50) to form a hetero-octameric complex [48]. 

PRMT5 is known for its ability to methylate histone and non-histone substrates. Among the histone substrates are H3, H2A, and H4, and non-histone substrates include p53, E2F-1 tumor suppressors, and kinases such as EGFR, BRAF, and transcription factors. Additionally, PRMT5 can form large complexes such as Swi/Snf or NCoR-SMRT [49]. 

PRMT5 overexpression can result in the activation of NF-kB, AKT, PI3K, and the mTOR/elF4E, contributing to cancer. Common cancers associated with the PRMT5 include lymphoma via the activation of the WNT/beta-catenin pathway, leukemia via suppression RB, DLBCL via upregulation of the BCR-BKT- NF-kB signaling, AML via regulation of alternative splicing by SRSF1 methylation, colorectal cancer via methylation of the BX1, Glioblastoma via the splicing of the ST7 tumor suppressor gene and bladder cancer via the enhancement of NF-kB activation [14]. 

PRMT5 inhibitors, namely SAM competitive inhibitors, SAM non-competitive inhibitors, and substrate-competitive inhibitors, have been utilized for cancer treatment. They are divided into first-generation inhibitors, which target the PRMT5 protein but result in adverse side effects like anemia and thrombocytopenia, and second-generation inhibitors that target the PRMT5-MTA complex which do not cause any severe side effects [14]. PRMT5 inhibitors are mostly effective against several kinds of carcinomas, such as solid tumors and diffuse large B-cell lymphoma, as well as non-Hodgkin’s lymphoma and AML [15]. 

The role PRMT5 plays in the development of breast cancer, lung cancer, prostate cancer, gastric cancer, pancreatic cancer, and melanoma is multifold. PRMT5 is overexpressed in breast cancer patients, and it results in the activation of the Wnt/beta-catenin proliferative signaling pathway, leading to enhanced expression of c-myc and CYCLIN D1 and SURVIVIN. The small molecule inhibitor CMP5 hinders PRMT5 function, causing diminished PRMT5 binding and histone methylation at the promoter regions of DKK1 and DKK3. Consequently, this leads to reduced expression levels of Cyclin D1 and SURVIVIN [49]. Treatments for triple-negative breast cancer have recently used the EPZ015666 PRMT5 inhibitors, which impair cell proliferation in this type of cancer [50]. In colorectal cancer, PRMT5 overexpression can result in the activation of NF- kB via arginine methylation on its p65 subunit, and anticancer therapies are based on targeting such interaction [51]. Glioblastoma is also associated with overexpression of PRMT5, which is correlated with cell growth rate. Initially, chemotherapy was the main form of treatment; however, currently, PRMT5 inhibitors show a significant growth inhibition on tumor cells, specifically the synthetic fragment-based MRTX9768, which binds to the PRMT5-MAT complex [52]. 

Over the past years, PRMT5 inhibitors have been utilized in clinical trials for solid and hematological malignancies [53]. PF-06939999 is an oral PRMT5 inhibitor that is used in endometrial, urothelial, cervical and esophageal cancers, as well as in NSCLC and head and neck SCC. The phase 1 clinical study concluded tolerable medication side effects and manageable safety profile [54]. PRT543 is a PRMT5 inhibitor used for solid tumors and lymphoma. It selectively binds and inhibits methyltransferase activity and in one case of homologous recombination deficiency ovarian cancer the response was durable. In 4 patients of ACC and one patient of uveal melanoma the cancer stabilized on this treatment [53]. A dose escalation trial of AMG 193 began in February 2022. AMG 193 is an oral MTA cooperative PRMT5 inhibitor used for non-small cell lung cancer and used in combination or IV docetaxel [53].

Two separate clinical trials are evaluating the selective small molecule inhibitor of PRMT5 GSK3326595 which works by inhibiting cellular mRNA splicing [55]. The first clinical trial concluded that 89% of the solid tumor cancer patients experienced drug-related adverse effects, namely fatigue, anemia, and nausea, and 41% of the patients needed dose reduction. The second clinical trial’s aim is to examine the safety of the drug for Myelodysplastic syndrome, chronic myelomonocytic leukemia, and hyperproliferative AML. A third trial will be evaluating the use of PRMT5 for breast cancer [53].

JNJ-64619178 is an irreversible PRMT5 inhibitor administered via oral capsule. It inhibits the S-adenosylmethionine and guanidino-binding pockets of PRMT5/MEP50 which translates to longer residence time, high binding affinity and extended stabilization [56]. The drug is most effective for tumors that display splicing instability. Stable disease was observed in 13% of the patients of the clinical trials which included patients with ACC, salivary and prostate cancer [53].

PRT811 is a selective PRMT5 that is used in high-grade gliomas such as glioblastoma or anaplastic astrocytoma. It works by crossing the blood–brain barrier and is particularly effective in uveal melanoma [53].

### 2.6. PRMT6

PRMT6 is a type 1 protein arginine methyltransferase located predominantly in the nucleus and found in regulatory DNA regions. It acts as a transcription inhibitor of cell cycle regulators such as CDKN1A, CDKN1B, CDKN2A and p53 [57], PRMT6 creates asymmetric dimethylation of histone H3R2, which serves as a repressor by opposing the trimethylation of H3 lysine 4 by MLL histone H3K4 methyltransferase. Overexpression of PRMT6 is observed in various cancers, such as prostate, cervical, bladder, and lung cancers, making it a potential target for therapeutic intervention [58]. 

PRMT6 is linked to gastric cancer through its ability to increase global levels of H3R2me2a, a histone modification associated with altered chromatin structure and gene expression patterns that promote cancer progression. Additionally, PRMT6 plays a crucial role in maintaining the pluripotency and self-renewal capabilities of embryonic stem cells (ESCs). Studies have demonstrated that reducing PRMT6 levels in ESCs results in a decrease in the expression of pluripotency-related genes and an increase in markers indicative of differentiation. While these findings highlight PRMT6’s potential as a therapeutic target, its clinical relevance in gastric cancer remains to be fully elucidated [59]. 

PRMT6 affects endometrial cancer by facilitation of EMC cell proliferation and migration via the AKT/mTOR signaling. PRMT6 is also associated with estrogen-dependent breast cancer as it plays a role in estrogen signaling. If inhibited, PRMT6 can restore global DNA methylation in MCF7. In lung cancer PRMT6 activates tumor associated macrophages via interaction with ILF2 [14]. 

In hepatocellular carcinoma, PRMT6 inhibits RAS/RAF bindings and MEK-ERK signaling. This action will suppress liver cancer stem cells and metastasis [60]. PRMT6 knockdown increases the androgen receptor pathway that is involved in the progression of prostate cancer providing potential clinical relevance [61]. 

Currently, there are limited active clinical trials investigating the therapeutic applications of PRMT6. Nonetheless, PRMT6 has potential as a diagnostic marker for colorectal cancer [62]. Furthermore, the PRMT6 inhibitor RPZ020411 has demonstrated efficacy in halting IL-6/STAT3 signaling, which is essential for the proliferation of breast cancer cells. This promising result warrants further evaluation of RPZ020411 for therapeutic purposes [63].

### 2.7. PRMT7

PRMT7, a type III arginine methyltransferase with dual S-Adenosyl-L-methionine (SAM) binding sites, plays pivotal roles in inflammation, DNA repair, cell cycle control, stress response, and stem cell proliferation [15,64,65,66,67,68,69,70]. Its unique monomethylation activity on arginine residues, especially notable in histone monomethylation within the NF-κB pathway in monocytes, aids in monocyte recruitment to injury sites [65]. This activity extends to DNA damage repair by modifying histone H4 at R3, crucial for DNA repair gene regulation via homologous recombination and nonhomologous end-joining pathways, influencing cellular responses to DNA-damaging agents like cisplatin and chlorambucil [15,66,67]. Despite this, PRMT7’s role in DNA repair remains complex, with some studies suggesting it induces sensitivity to DNA damage [68,69]. 

PRMT7’s regulation of histones also affects muscle regeneration, promoting cell-cycle arrest and hindering myofiber renewal [66]. It has been demonstrated that PRMT7 enforces cell-cycle arrest at the G1 phase by the suppression of cyclin-dependent kinases, mediated through the stabilization and accumulation of p21 [67]. Notably, a whole-body knockout mouse model elucidated the importance of PRMT7 in maintaining satellite cells, which are essential for muscle regeneration [66]. Additionally, PRMT7 plays roles in stress response and stem cell growth, with deficiencies leading to developmental abnormalities [70,71]. Mutations in PRMT7 are linked to a genetic disorder characterized by a short stature and intellectual disability, among other symptoms [72,73].

In cancer research, PRMT7 is of significant interest due to its varying expression across several cancers, including prostate cancer, breast cancer, melanoma, non-small-cell lung cancer (NSCLC), gastric cancer, T-ALL, liver cancer, and renal cell carcinoma. PRMT7 expression is increased in prostate cancer tumors with further expression in metastatic tumors, although some datasets provided conflicting results [74]. Its overexpression in metastatic breast cancer and interaction with oncogenes suggest a role in cancer progression, possibly through mechanisms like MMP9 upregulation and E-cadherin inhibition, which promotes cell invasion and epithelial-to-mesenchymal transition [75,76,77,78,79].

Notably, PRMT7’s expression in melanoma inversely correlates with patient survival, where it modulates immune recognition through histone modification, affecting IFN gene expression and MHC-I levels on tumor cells. This mechanism highlights PRMT7’s potential in immunotherapy, where its deletion enhances tumor cell recognition and destruction by upregulating MHC-I on the surface of melanoma cells [80].

In NSCLC, T-ALL, and renal cell carcinoma, PRMT7’s overexpression is associated with increased invasion and poor prognosis [81,82,83]. In NSCLC, PRMT7’s overexpression promotes cancer cell invasion by way of HSPA5 and EEF2 proteins [81]. In T-ALL, depleting PRMT7 caused reduced cell viability by decreasing RUNX1 methylation and thus downregulating its target gene expression [82]. PRMT7 expression was correlated with a poor prognosis in renal cell carcinoma by upregulating c-Myc expression through methylation (and stabilization) of β-catenin [83]. On the other hand, decreased PRMT7 expression is linked to tumorigenesis in liver and gastric cancers, mediated through interactions with miR-24-2 and PTEN, respectively [84,85]. In general, it has been suggested that PRMT7 may influence various cancers through its methylation [86].

PRMT7 inhibitors have shown promise in enhancing current chemotherapy treatments [67]. In a breast cancer cell line, MCF7, a PRMT7 inhibitor combined with doxorubicin had synergistic effects on cytotoxicity [67]. Additionally, genetic loss of PRMT7 induced reprogramming of glycine metabolism that eradicated leukemic stem cells in mouse and human models of chronic myeloid leukemia (CML) [87]. This deletion increased survival, reduced splenomegaly, and tumor burden significantly [87]. Finally, in a mouse model of melanoma, inhibition of PRMT7 combined with anti-CTLA-4 and anti-PD-1 treatments led to better suppression of tumor growth [80]. These findings advocate for further exploration into PRMT7 as a therapeutic target across various malignancies, taking care to consider its dual roles in cancer progression and suppression.

### 2.8. PRMT8

PRMT8 is the only membrane-bound PRMT and its expression is limited to the neurons of the central nervous system (CNS) [88,89]. Among its unique structural features are an N-terminal myristoylation site that allows it to adhere to the membrane, and a proline rich sequence on the N-terminus that facilitates its interaction with PRMT2 and SH3 domain-containing proteins [88]. PRMT8 is negatively regulated by automethylation [90]. In the dendritic spines of neurons, PRMT8 modulates synaptic actin dynamics, crucial for the growth of Purkinje cells and synaptic functionality [89,91]. 

PRMT8 contributes to stress tolerance in motoneurons, preventing degeneration in spinal cord injury (SCI) and amyotrophic lateral sclerosis (ALS) [92,93,94]. Mechanistically, PRMT8 increases mitochondrial stress capacity, stabilizes phospholipids, and decreases inflammation [95]. Loss of PRMT8 activity leads to DNA damage accumulation and neurodegeneration, while its overexpression can mitigate pro-inflammatory responses and safeguard neurons against SCI-induced damage [92,93]. Similarly, PRMT8 is protective in the pathogenesis of fused in sarcoma-associated ALS [94]. 

Despite these insights, the implications of PRMT8 expression in CNS malignancies remain ambiguous, with studies presenting conflicting evidence. For instance, PRMT8 has been identified as necessary for the proliferation of grade IV glioblastoma cells in some research, whereas another study suggests its depletion might contribute to glioma progression [96,97]. 

Beyond the nervous system, PRMT8’s expression and its potential pathogenic roles in various non-neuronal cancers, including breast, cervical, and prostate cancers, are still under exploration [88,98]. The relationship between PRMT8 expression and cancer prognosis varies across studies and cancer types. In breast and ovarian cancers, high PRMT8 levels have been associated with improved patient survival, although no increased PRMT8 expression was observed in triple-negative breast cancer [21,98]. A third study demonstrated conflicting results regarding PRMT8’s ability to stabilize or protect against breast cancer tumors, urging further research to clarify its role in specific cancer subtypes [99]. Conversely, elevated PRMT8 expression correlates with poorer outcomes in gastric cancer patients and appears to foster tumor growth in colon cancer [98,100]. 

Given these findings, PRMT8’s multifaceted role across neural protection, neurodegeneration, and cancer presents a complex picture that necessitates more detailed investigations to fully understand its therapeutic potential and implications in cancer pathogenesis.

### 2.9. PRMT9

PRMT9 plays a role in normal neural development by acting in RNA splicing. It does so by methylating its substrate, splicing factor 3B subunit 2 (SF3B2) [101]. PRMT9 has also been shown to interact with the spliceosome-associated proteins SAP145 and SAP49, which are constituents of U2snRNP. PRMT9 has been shown to have a remarkably high affinity for its substrates, differentiating it from the majority of the other PRMT proteins, which do not have as strong of an affinity for their respective substrates [102].

PRMT9 has been shown to act as an oncogene in the development of acute myeloid leukemia (AML). Studies using both PRMT9 knockout cells and PRMT9 inhibitors demonstrated that targeting this protein not only inhibits the formation of oncoproteins but also activates an intrinsic immune response from T-cells via a type I IFN response. The underlying mechanism of this immune response is dependent on cyclic GMP-AMP synthase activation [103]. PRMT9 has also been shown to have increased expression in certain skin cancers, testicular cancers, pancreatic cancers, lymphomas, and hepatocellular carcinoma (HCC). Both in vitro and in vivo studies have demonstrated a possible link between increased PRMT9 expression and metastasis of HCC to other tissues, namely lung metastases. This study hypothesizes a mechanism for the oncogenic effects of PRMT9 to be through its activation of the PI3K/Akt/ GSK-3b/Snail pathway, resulting in an observed increase in Snail expression and decrease in E-cadherin expression [104]. These studies highlight the importance of further research and analysis of PRMT9 as an oncogene and its potential value as a clinical biomarker for numerous cancers.

A compound referred to as LD2 has been shown via assays with AML cell lines to be a potent inhibitor of PRMT9. The results of the study included a decrease in cancer cell viability while simultaneously increasing the number of T-cells. In addition, the anticancer effects of LD2 have been shown to be enhanced when used in combination with PD-1 monoclonal antibody treatment. AML-afflicted mice models treated with this combination of treatments resulted in decreased cancer cell proliferation and increased survival rates. These results warrant further investigation into the concurrent usage of PRMT9 inhibitors with other pharmaceuticals for AML treatment [103]. In addition, a selective and potent inhibitor of PRMT9, called EML1219, has been synthesized. This compound has been shown to have a KD of 188 nM and a preferential selectivity for PRMT9 [105]. Likewise, these results warrant future studies using in vivo models for the investigation of the use of EML1219 for clinical purposes.

## 3. Conclusions

PRMTs are a class of enzymes involved in epigenetic regulation for various cellular functions through arginine methylation. PRMT dysregulation and overexpression has been demonstrated in various cancer types and is correlated with poor patient prognosis. Various PRMT inhibitors have been developed and have shown therapeutic promise. In this review, we underscored the importance of further research into PRMTs to fully elucidate their mechanisms and the further development, testing, and optimization of novel compounds targeting PRMTs, as this has significant therapeutic potential for cancer patients. Further research should also be conducted to explore biomarker development for patient stratification, as well as combination therapeutic strategies with PRMT inhibitors, such as with Pluvicto, as combination therapies can show significant therapeutic effects for cancer patients [106]. An overview of the major cancer associations of PRMT family members and their lead small-molecule inhibitors can be seen in Table 3 below.

## Figures and Tables

**Table 1:** Mechanism of action of PRMT family members.

PRMT	Type	Mechanism of Action
**PRMT1**	I	Transcription activation; cGAS–STING methylation; Drives proliferation/survival
**PRMT2**	I	Nuclear co-regulator; Modulates Rb/E2F and NF-κB programs
**PRMT3**	I	Ribosomal RPS2 methylation; Boosts translation and glycolysis (LDHA)
**PRMT4 (CARM1)**	I	H3R17/26 methylation; Transcriptional activation; AKT/mTOR signaling
**PRMT5**	II	SDMA on Sm proteins; Splicing dependency; PI3K/AKT, mTOR/eIF4E
**PRMT6**	I	H3R2me2a opposes H3K4me3; Represses CDK inhibitors
**PRMT7**	III	MMA marks; DNA-damage response; EMT and immune modulation
**PRMT8**	I (mem-bound)	Membrane-anchored paralog; Enhances migration/invasion; Therapy tolerance
**PRMT9**	II	SF3B2 methylation; Splicing rewiring; AKT–GSK3β–Snail EMT

**Table 2:** Cancer-Relevant Roles of The PRMT Family Members.

PRMT	Type	Cancer Relevant Roles
**PRMT1**	I	Oncogenic signaling; immune evasion; supports proliferation
**PRMT2**	I	Modulates Rb/E2F, NF-κB; promotes proliferation/migration
**PRMT3**	I	Metabolic rewiring (LDHA, glycolysis); tumor growth and invasion
**PRMT4 (CARM1)**	I	Activates AKT/mTOR; blocks myeloid differentiation
**PRMT5**	II	Oncogenic signaling (NF-κB, PI3K/AKT, mTOR/eIF4E)
**PRMT6**	I	H3R2me2a-mediated transcriptional repression; impacts AKT/mTOR
**PRMT7**	III	DNA-damage response; immune modulation; EMT/invasion programs
**PRMT8**	I (mem-bound)	Promotes proliferation/migration/invasion; drug tolerance
**PRMT9**	II	Drives EMT/invasion and metastasis via PI3K–AKT–GSK-3β–Snail signaling

**Table 3:** Major Cancer Associations of The PRMT Family Members and Their Lead Small-Molecule Inhibitors.

PRMT	Type	Major Cancer Associations	Lead Small-Molecule Inhibitors (Stage)
**PRMT1**	I	Breast, lung, AML	AMI-1, MS023, GSK3368715 (phase I)
**PRMT2**	I	RCC, HCC, breast, glioblastoma	—
**PRMT3**	I	Glioblastoma, colorectal, pancreatic, HCC	SGC707 (preclinical)
**PRMT4 (CARM1)**	I	HCC, AML, multiple myeloma	TP-064 (preclinical); Compound 49 (preclinical)
**PRMT5**	II	TNBC, colorectal, glioblastoma, hematologic malignancies	GSK3326595 (phase I/II), JNJ-64619178 (phase I), PF-06939999 (phase I)
**PRMT6**	I	Gastric, prostate, breast, lung	RPZ020411 (preclinical)
**PRMT7**	III	Breast, melanoma, NSCLC, CML	SGC8158 (preclinical)
**PRMT8**	I (mem-bound)	Glioblastoma; roles mainly neuro-protective	—
**PRMT9**	II	AML, HCC, metastatic skin/testis tumors	EML1219 (preclinical); LD2 (preclinical)

## Abbreviations

The following abbreviations are used in this manuscript:

PRMT5	Protein arginine methyltransferase 5
CRC	Colorectal Cancer
COAD	Colon adenocarcinoma
ERK	Extracellular signal-regulated kinase
KRAS	Kirsten rat sarcoma viral oncogene homolog
READ	Rectum adenocarcinoma
RCC	Renal Cell Carcinoma
